# Effects of Alternative Food Sources and Different Substrates on the Mass Rearing of *Amblyseius andersoni*

**DOI:** 10.3390/plants14182912

**Published:** 2025-09-19

**Authors:** Angelos Bechtsoudis, Maria L. Pappas, Konstantinos Samaras, George D. Broufas

**Affiliations:** Laboratory of Agricultural Entomology and Zoology, Department of Agricultural Development, Faculty of Agricultural and Forestry Sciences, Democritus University of Thrace, 68200 Orestiada, Greecempappa@agro.duth.gr (M.L.P.);

**Keywords:** biological control, factitious food, mass rearing, phytoseiids, pollen, prey, tomato

## Abstract

The predatory mite *Amblyseius andersoni* Chant (Acari: Phytoseiidae) is a key biological control agent against spider mites and other pests. For its broad application, efficient and affordable mass-rearing systems are essential. This study evaluated the effects of rearing substrate, food type, and rearing history on the development, survival, reproduction, and predation efficiency of the predator. Mites were reared on leaf discs or Plexiglas plates and fed one of five diets, including various plant pollens and the stored product mite *Carpoglyphus lactis* (L.) (Acari: Carpoglyphidae). Additionally, it was assessed whether rearing five generations on cattail pollen supplemented with the natural prey (*Tetranychus urticae* Koch (Acari: Tetranychidae) or frozen *C. lactis* influenced later predators’ performance. The substrate type did not affect development or survival contrary to the food source, with mites fed on cattail pollen or *C. lactis* developing faster and producing more eggs. Survival remained high across all diets. The intrinsic rate of increase was highest with cattail pollen and *C. lactis*. The five-generation rearing did not affect performance or feeding on natural prey such as *T. urticae* or *Aculops lycopersici* (Tryon) (Acari: Eriophyidae). These findings demonstrate that *A. andersoni* can be effectively mass-reared on alternative diets and substrates, supporting biocontrol strategies.

## 1. Introduction

Integrated pest management (IPM) is an important pest control strategy, aiming to reduce dependence on chemical pesticides while promoting environmentally friendly, sustainable solutions for pest suppression [1,2]. Biological control with the use of natural enemies to regulate pest populations, is a key component of IPM that has received considerable attention lately [3,4,5]. Among the most efficient biological control agents are predatory mites of the family Phytoseiidae, which are routinely used to manage key pests such as herbivorous mites and insects in several crops. Their fast development, high reproductive potential, and predation efficiency make them ideal candidates for both preventive and curative biological control [6,7,8].

Predatory mites can be released in various cropping systems such as open-field crops, orchards, and greenhouses [9,10], where phytoseiid mites are most successfully used. To meet producers’ demands, commercial rearing of phytoseiid mites is supported by improvements in mass-rearing techniques and the increasing acceptance of biocontrol in IPM systems [2,11]. However, along with the growing demand for biocontrol agents, maintaining their quality and reliability during mass production is a significant challenge.

Among phytoseiid mites, *Amblyseius andersoni* (Chant) (Acari: Phytoseiidae) is a generalist predator that is widely distributed across North America, Europe, and the Mediterranean basin. It can thrive in diverse habitats and climates [10,12,13,14,15,16]. According to McMurtry et al. [17], *A. andersoni* is classified as a type III generalist predator with a broad prey range, including plant-feeding mites (Eriophyidae, Tarsonemidae, Tetranychidae, Tenuipalpidae), as well as soft-bodied insects like thrips and whiteflies. It is commercially available in greenhouse crops and orchards for biological control of spider mites and thrips [11,12]. In orchards and vineyards, *A. andersoni* is commonly associated with *Panonychus ulmi* Koch (Acari: Tetranychidae) and *Tetranychus urticae* Koch (Acari: Tetranychidae) [18,19,20]. In the absence of prey or during periods of prey scarcity, *A. andersoni* can feed and reproduce on various alternative food sources, including pollen, factitious prey species, or fungal spores [14,21,22,23,24,25,26,27]. This feeding behavior ensures its persistence in the crop, making this predator particularly suitable for inoculative or preventive releases.

A critical aspect of the successful mass rearing of biological control agents is the provision of adequate nutritious food sources [24]. While natural prey, such as *T. urticae*, is of high nutritional value and supports predator performance, maintaining pest colonies is resource and labor-intensive. In contrast, alternative food sources such as pollen and factitious prey offer advantages in terms of availability, cost, and ease of handling [24]. Pollen, in particular, has been shown to support the development and reproduction of many generalist phytoseiids, including *A. andersoni*, *Amblyseius swirskii* Athias-Henriot (Acari: Phytoseiidae), and *Neoseiulus cucumeris* (Oudemans) (Acari: Phytoseiidae), etc. [17,18,28,29,30,31,32,33,34,35]. Pollen not only serves as a supplemental or alternative food source in the absence of prey but can also sustain predator populations during periods of low prey availability [27,36]. For example, pollen from *Typha angustifolia* L. (Typhaceae) has been successfully used in both laboratory and commercial settings [27]. However, different pollen types vary in nutritional composition and physical characteristics, influencing their suitability for particular predator species [30].

Apart from pollen, several species of astigmatid mites, such as *Tyrophagus putrescentiae* (Schrank) (Acari: Acaridae) [37], *Carpoglyphus lactis* (Lineaus) (Acari: Carpoglyphidae) [38,39], *Suidasia medanensis* Oudemans (Acari: Suidasiidae) [40] and *Lepidoglyphus destructor* (Schrank) (Acari: Glycyphagidae) [41], have been tested and shown to be valuable alternative food sources for the mass rearing of generalist phytoseiid mites, due to their low production costs and high nutritional value. In some cases, astigmatid mites have been directly offered on plants or provided in sachets to facilitate the early establishment of phytoseiid predators in greenhouse conditions where prey is absent [7,27,42,43,44,45]. Notably, among the astigmatic and factitious prey species, *C. lactis* has been tested with good results in the rearing of predatory phytoseiids, either as a standalone food source or often in combination with specific pollens to improve nutritional balance and boost predator population growth [39,46,47,48,49].

Recent studies have also demonstrated differences between frozen and live astigmatic prey for phytoseiid predators. For instance, *A. swirskii* had higher oviposition rates and faster juvenile development when fed frozen *T. putrescentiae* compared to live individuals [43]. Similarly, *Transeius montdorensis* (Schicha) (Acari: Phytoseiidae) showed enhanced fecundity when fed on pollen supplemented with frozen compared to live eggs of *C. lactis* [50]. These findings emphasize the importance of prey status on predator performance. While frozen prey provides practical benefits, such as easier handling, longer shelf life, and reduced contamination risk, live prey may better encourage natural foraging and feeding behavior. Therefore, optimizing the use of live and/or frozen astigmatids, possibly combined with supplements such as pollen is crucial for enhancing predator quality and maintaining the cost-effectiveness of the mass-rearing system. On the other hand, relying too heavily on such live or frozen factitious prey in mass-rearing setups can significantly impact the quality and behavior of the mass-reared predators, reducing their performance, even over a short period, as reported in the case of *Amblyseius orientalis* Ehara (Acari: Phytoseiidae) [51].

Besides diet, substrate type where mass rearing occurs can influence key life-history traits of biological control agents [50]. Leaf discs (or plants) mimic natural microhabitats and may encourage natural movement and oviposition behavior of predatory mites. Conversely, artificial surfaces like Plexiglas plates are durable, easy to clean, and standardized for experimental setups or production systems, but they may hinder some behaviors or decrease survival [46]. Still, some studies have shown they can increase fecundity under certain conditions [52]. Understanding how rearing substrates interact with food sources is crucial for optimizing predator performance and mass-rearing systems.

Despite the use of alternative diets and artificial substrates in mass rearing [53], relatively few studies have examined their interactive effects on the development, survival, and fecundity of phytoseiid mites. Moreover, short-term laboratory studies may overlook the cumulative effects of rearing conditions when maintained over multiple generations. Long-term rearing on artificial diets or suboptimal conditions can lead to reduced fitness, altered prey preferences, or shifts in reproductive traits that could compromise predator efficiency in the field [46]. Monitoring such changes across generations is essential for ensuring that mass-reared populations retain their biological control potential [54,55].

To address these knowledge gaps, the present study investigated the effects of various food sources and rearing substrates on the performance of *A. andersoni*. Specifically, we assessed the impact of several pollen types and the factitious prey *C. lactis* (frozen individuals) on the development, survival, and oviposition, in combination with two rearing substrates: leaf discs and Plexiglas plates. In a second experiment, we examined the effects of mass rearing for five consecutive generations on two different diets: (i) frozen *C. lactis* individuals plus pollen and (ii) *T. urticae* plus pollen, focusing on development time, survival rate, oviposition, and prey consumption. This study aims to contribute to the short- and long-term effects of diet and rearing conditions on the performance of *A. andersoni*, with practical relevance for improving the quality of its mass rearing.

## 2. Results

### 2.1. Effects of Different Substrates and Food Sources on Predator Performance

**Figure 1:**
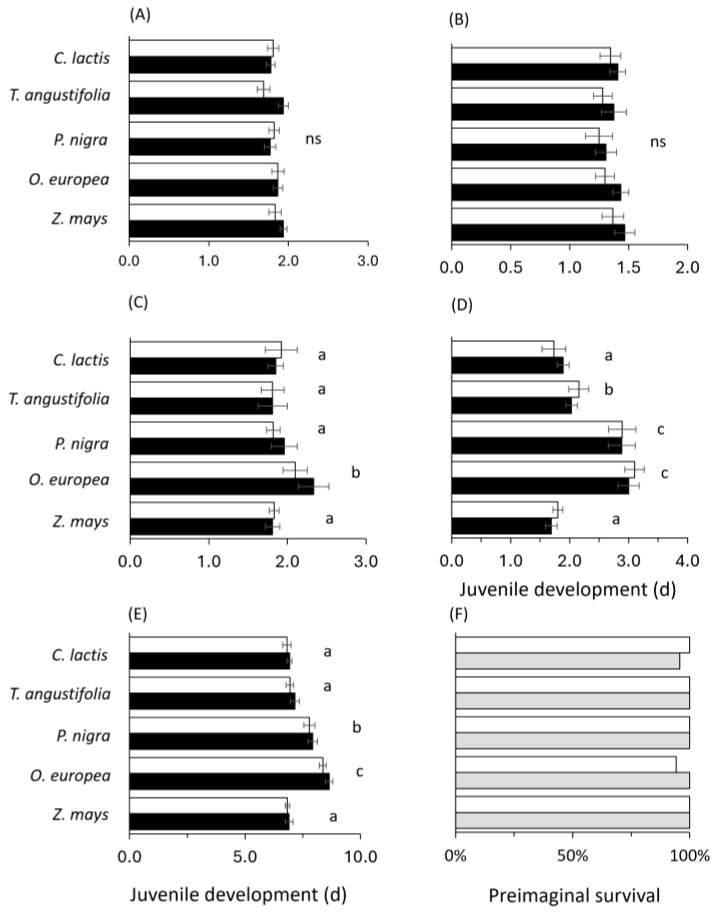
Effects of different food sources on the developmental time of *Amblyseius andersoni*: (**A**) egg, (**B**) larvae, (**C**) protonymph, (**D**) deutonymph, and (**E**) egg to adult (males: white bars, females: black bars) and (**F**) juvenile survival (leaf: grey bars; Plexiglas: white bars). Mites were reared on either tomato leaves or Plexiglas; however, since the effect of substrate was not significant, the data were pooled. Experiments were conducted at 25 ± 1 °C and 16:8LD. Means in the same group (developmental stage and food) followed by different lowercase letters are significantly different (marginal means were compared using the Least Significant Difference LSD). ns = not significant.

The rearing substrate (leaf disc vs. Plexiglas) did not significantly affect the developmental time per instar or the total juvenile developmental time, regardless of the food source or sex of the predatory mite. No significant main effects or interactions involving the substrate were observed (Substrate: 0.069 ≤ Wald χ^2^ ≤ 3.044; 0.227 ≤ *p* ≤ 0.856; Substrate × Sex: 0.006 ≤ Wald χ^2^ ≤ 0.006; 0.543 ≤ *p* ≤ 0.939; Substrate × Food: 0.363 ≤ Wald χ^2^ ≤ 8.396; 0.078 ≤ *p* ≤ 0.985; Substrate × Food × Sex: 0.886 ≤ Wald χ^2^ ≤ 8.741; 0.068 ≤ *p* ≤ 0.927). Therefore, data from the two substrates were pooled for further analysis, as presented in Figure 1A–D. The effect of sex was also found to be non-significant across all developmental instars (Sex: 0.069 ≤ Wald χ2 ≤ 2.749; 0.097 ≤ *p* ≤ 0.793).

Among the factors tested, only the food source had a significant effect on the total developmental time (Wald χ^2^ = 10.336; df = 4; *p* = 0.035) and those of the protonymph (Wald χ^2^ = 137.278; df = 4; *p* ≤ 0.001) and deutonymph stages (Wald χ^2^ = 158.836; df = 4; *p* ≤ 0.001). Predators reared on *O. europea* pollen showed the longest developmental time, followed by those reared on *P. nigra* pollen, with significantly shorter duration recorded for the other food sources (Figure 1E), and a similar trend in the protonymph (Figure 1C) and the deutonymphal instar (Figure 1D). The survival of the juvenile predators was similar on the different food sources, and did not differ among those reared on leaf discs over Plexiglas, ranging from 94 to 100% (χ^2^ = 5.99; df = 9) (Figure 1F).

**Figure 2:**
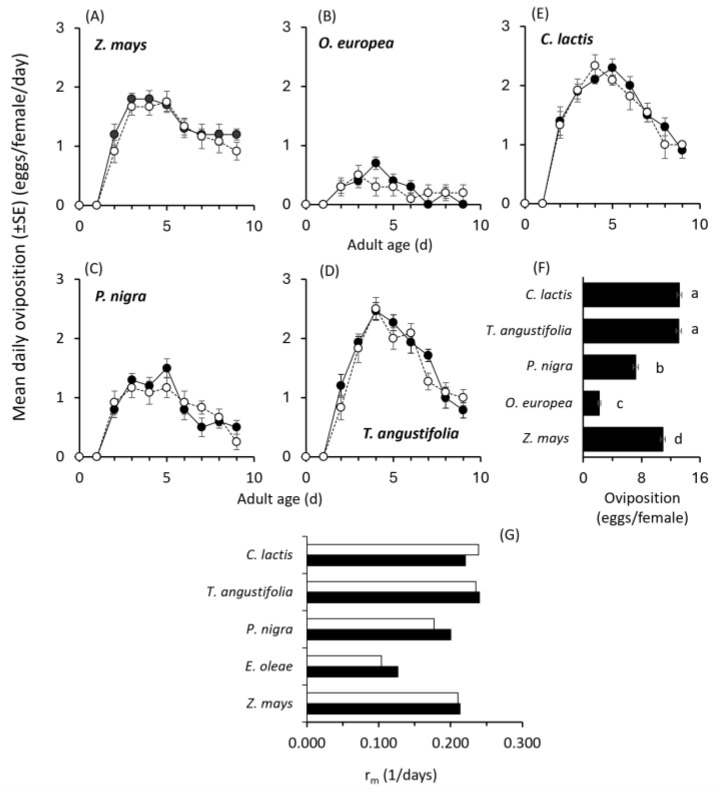
Effects of different food sources on daily oviposition (**A**–**E**) of *Amblyseius andersoni* reared either on tomato leaf (black dots) or Plexiglas (white dots) arenas, respective cumulative oviposition over one week (**F**) on both substrates (data combined since the effect of substrate was not significant), and (**G**) on the intrinsic rate of increase (*r_m_*) for mites reared either on tomato leaf (black bars) or Plexiglas (white bars). Experiments were conducted at 25 ± 1 °C and 16:8 LD. Means in the same group followed by the same letter are not significantly different (marginal means were compared using the Least Significant Difference LSD).

Furthermore, mean daily oviposition curves were similar within each food source independently of the rearing substrate (Figure 2A–E), whereas cumulative oviposition per female was significantly affected (Food (F): Wald χ^2^ = 157.422; df = 4; *p* < 0.001; Substrate: Wald χ^2^ = 0.390; df = 1; *p* = 0.532; Sub × F: Wald χ^2^ = 0.194; df = 4; *p* = 0.996, Figure 2F). The recorded mean cumulative oviposition was significantly higher for mites reared on cattail pollen or *C. lactis* followed by *Z. mays* and *P. nigra* pollen, with the lowest number recorded for *O. europea* pollen (Figure 2F).

For each food source, the calculated *r_m_* values were similar between the two rearing substrates (leaf disc vs. Plexiglas (Figure 2G). Among the different food sources, the highest *r_m_* values recorded were those for the cattail pollen and *C. lactis* individuals (ranging between 2.22 and 2.24).

### 2.2. Mass-Rearing Effects on Predator Performance

**Figure 3:**
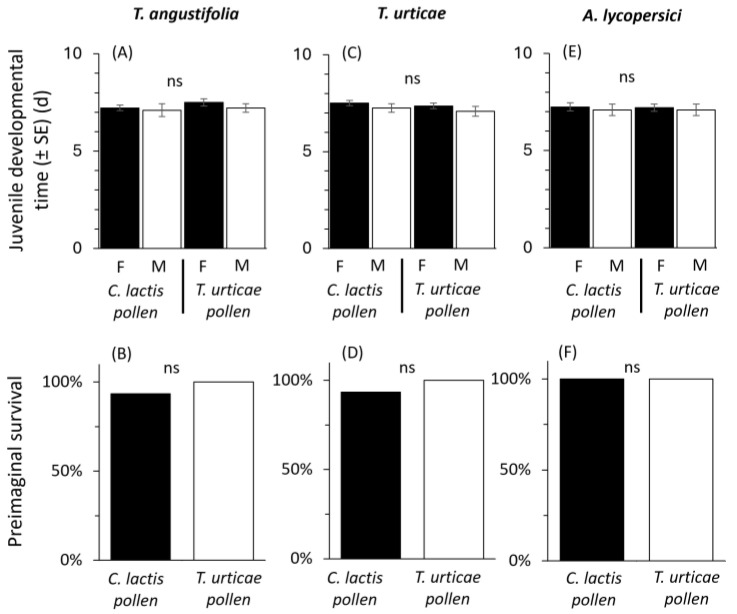
Effects of rearing *Amblyseius andersoni* for five consecutive generations on a factitious (frozen *Carpoglyphus* lactis) vs. prey (*Tetranychus urticae*) species, both supplemented with cattail pollen on: juvenile developmental time of males (white bars) and females (black bars), and respective juvenile survival (%) when mites were reared on *Typha angustifolia* pollen (**A**,**B**), *Tetranychus urticae* (**C**,**D**), or *Aculops lycopersici* (**E**,**F**). ns = not significant.

For both *A. andersoni* populations that were reared for five generations either on spider mites or *C. lactis*, plus cattail pollen, total juvenile developmental time was similar and not significantly differentiated by the rearing history of the colony, the sex, or their interaction when mites were reared on *T. angustifolia* pollen (Rearing history (H): Wald χ^2^ = 1.002; df = 1; *p* = 0.317; Sex(S): Wald χ^2^ = 1.085; df = 1; *p* = 0.298; H × S: Wald χ^2^ = 0.174; df = 1; *p* = 0.676, Figure 3A), young larvae of *T. urticae* (H: Wald χ^2^ = 0.843; df = 1; *p* = 0.359; S: Wald χ^2^ = 2.340; df = 1; *p* = 0.126; H × S: Wald χ^2^ = 0.010; df = 1; *p* = 0.919, Figure 3C) or *A. lycopersici* (H: Wald χ^2^ = 0.007; df = 1; *p* = 0.935; S: Wald χ^2^ = 0.365; df = 1; *p* = 0.546; H × S: Wald χ^2^ = 0.007; df = 1; *p* = 0.935, Figure 3E). Mean total developmental time was approx. 7.3 days. Similarly, juvenile survival was high (93–100%) and not affected by the use of astigmatic over spider mites in the predator’s diet (χ^2^ = 1.03, df = 1; Figure 3B,D,F).

**Figure 4:**
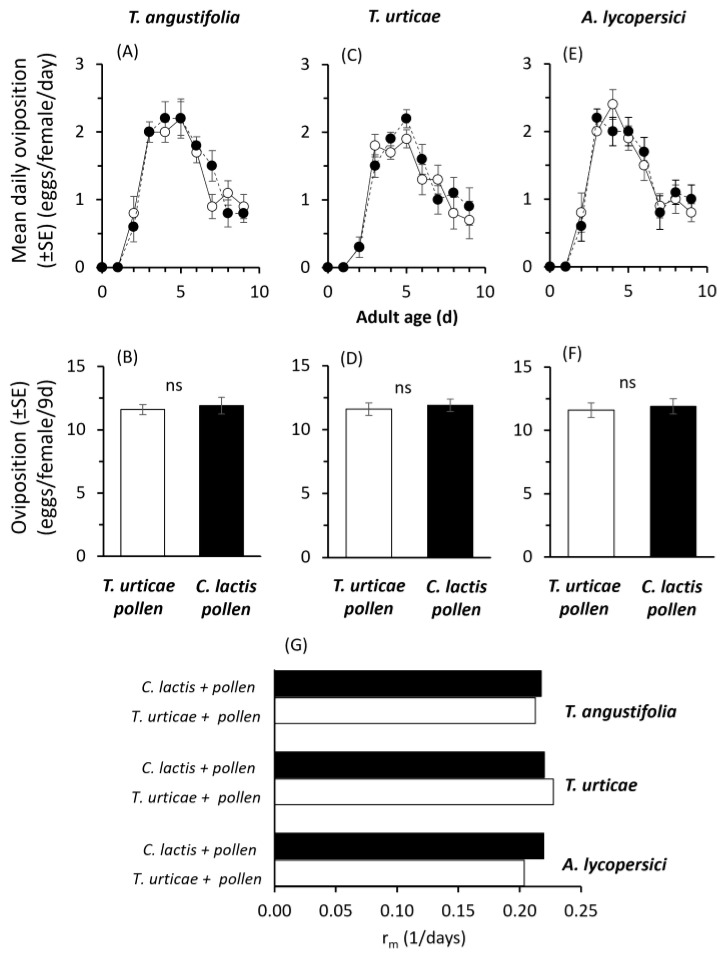
Effects of rearing *Amblyseius andersoni* for five consecutive generations on a factitious (frozen *Carpoglyphus lactis*) (black bars/dots) vs. prey (*Tetranychus urticae*) (white bars/dots) species, both supplemented with cattail pollen on: (i) daily, the (ii) respective cumulative oviposition on *Typha angustifolia* pollen (**A**,**B**), *Tetranychus urticae* (**C**,**D**), or *Aculops lycopersici* (**E**,**F**), (iii) on the intrinsic rate of increase (*r_m_*) (G). Experiments were conducted at 25 ± 1 °C and 16:8LD; ns = not significant.

The mean daily oviposition patterns on the three tested food sources (two prey species and cattail pollen) were similar between the two *A. andersoni* populations (Figure 4A,C,E). The corresponding cumulative oviposition per female ranged from 9.8 to 11.4 eggs/female and did not differ significantly between populations when reared on cattail pollen (t = 0.390, df = 18, *p* = 0.701; Figure 4B), *T. urticae* (t = 1.00, df = 18, *p* = 0.331; Figure 4D), or *A. lycopersici* (t = 0.120, df = 18, *p* = 0.906; Figure 4F).

Similarly, the estimated intrinsic rates of increase were comparable between the two predator populations reared on either *T. urticae* or frozen astigmatic mites, across all food sources: cattail pollen (0.213–0.218 d^−1^), *T. urticae* (0.204–0.219 d^−1^), and *C. lactis* (0.227–0.220 d^−1^) (Figure 4G).

**Figure 5:**
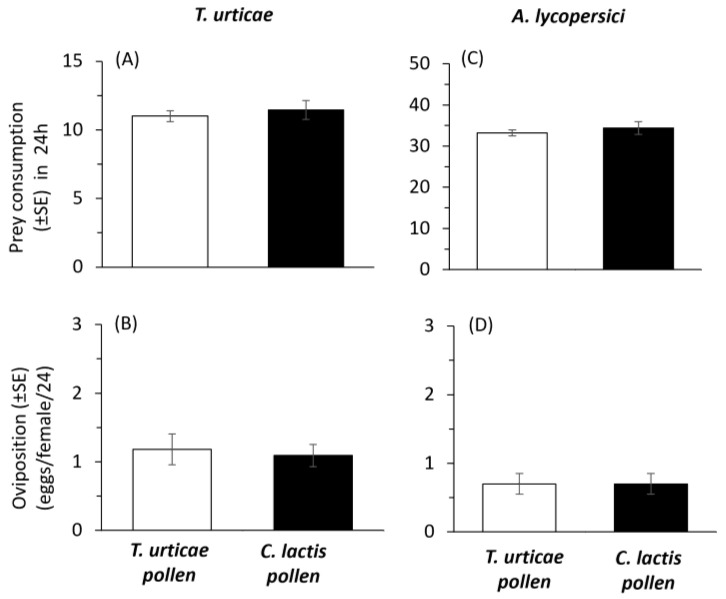
Effects of rearing *Amblyseius andersoni* for five consecutive generations on a factitious (frozen *Carpoglyphus lactis*) (black bars) vs. prey (*Tetranychus urticae*) (white bars) species, both supplemented with cattail pollen, on: (i) mean daily consumption, and (ii) oviposition when fed on *Tetranychus urticae* larvae (**A**,**B**), or *Aculops lycopersici* (**C**,**D**). Experiments were conducted at 25 ± 1 °C and 16:8LD.

Finally, the estimated mean daily prey consumption did not differ significantly between *A. andersoni* populations that were mass-reared on the two diets when fed on either *T. urticae* (t = 0.567, df = 20, *p* = 0.557) or *A. lycopersici* (t = 0.714, df = 18, *p* = 0.484), with consumption rates ranging from approx. 11.0–11.4 to 33.2–34.4 prey individuals per predator, respectively (Figure 5A,B).

The recorded mean daily oviposition rates for the two *A. andersoni* populations were similar on both prey species. On *T. urticae*, females laid approximately 1.1 eggs per day, with no significant difference between treatments (t = 0.326, df = 20, *p* = 0.748) (Figure 5B). Similarly, oviposition on *A. lycopersici* averaged around 0.9 eggs per female per day, also showing no significant difference (t = 0.372, df = 18, *p* = 0.714) (Figure 5D).

## 3. Discussion

The present study aimed to evaluate the performance of the generalist predatory mite *A. andersoni* when reared on various food sources, including natural prey and factitious diets, on two rearing substrates. We also investigated whether a five-generation rearing history on a factitious diet (i.e., *C. lactis*) affects predator fitness when returned to different food sources. Our results show that the choice of rearing substrate (leaf disc vs. Plexiglas) did not significantly influence predator performance. Food source, however, had a pronounced effect, with *T. angustifolia* pollen and frozen *C. lactis* supporting superior performance. No significant deterioration in fitness traits was observed for predators reared on the factitious food over five generations, suggesting that short-term mass rearing on such diets does not compromise predatory potential. These findings are relevant for both basic ecological understanding and applied biocontrol programs.

Our initial hypothesis was that rearing substrate might influence predator performance due to potential differences in microhabitat characteristics, especially humidity retention or opportunities for plant feeding by the mites. Leaf discs, as a natural substrate, could offer improved conditions or feeding opportunities, which might be beneficial for development or survival, as previously reported for other phytoseiids for instance, *Iphiseius degenerans* (Acari: Phytoseiidae) which when reared on leaf arenas (bean or pepper) exhibited significantly longer development times and improved juvenile survival compared to individuals on artificial substrates such as Multicel [56]. However, *A. andersoni* did not exhibit differential performance across the two substrates, a result that simplifies the logistics of rearing and experimentation, as artificial surfaces such as Plexiglas are easier to use and standardize.

The food source provided had a strong effect on developmental rate, oviposition, and the intrinsic rate of increase. Predators reared on *O. europaea* and *P. nigra* pollen exhibited slower development and lower fecundity compared to those fed cattail (*T. angustifolia*) pollen or *C. lactis*. These results suggest significant differences in the nutritional quality of plant pollen, supporting previous findings [32,34,50,57]. The poor performance on olive pollen highlights the importance of selecting high quality alternative foods to support predatory mite populations. Although we did not perform a biochemical analysis of the tested pollens, earlier studies have indicated that the protein, lipid, and starch composition, along with grain size and digestibility, are critical factors influencing pollen suitability [58,59,60,61].

The factitious food, frozen *C. lactis*, supported high intrinsic rates of increase, similar to those of cattail pollen. This is an important finding, as *C. lactis* is already used for mass rearing other phytoseiids such as *A. swirskii* and *N. cucumeris* [38]. Our results extend this suitability to *A. andersoni*, confirming the predator’s ability to exploit this food source. Moreover, our findings contribute to the growing body of literature supporting the use of astigmatic mites as cost effective alternatives to live prey in mass-rearing systems [39,47,49,50,62].

Regarding the potential impact of mass rearing on predator performance, we hypothesized that feeding on a factitious diet over multiple generations might reduce predator performance on natural prey due to nutritional imbalances or loss of prey handling experience. However, the five-generation rearing period on frozen *C. lactis* and cattail pollen did not lead to any measurable deterioration in fitness, as evaluated through development time, oviposition, intrinsic rate of increase, and prey consumption on *T. urticae* and *A. lycopersici*. These results suggest that short-term rearing on factitious diets does not impair predator performance. Even though in a similar study, six generations on factitious food (almond pollen) were adequate to significantly affect the quality of mass-reared *A. swirskii* [63]; more extended rearing periods are generally thought to be necessary to fully identify diet-induced changes in demographic parameters, prey preference, or physiological adaptations [64]. However, such effects may depend on species and diet-specific characteristics. For example, rearing *Neoseiulus californicus* (McGregor) (Acari: Phytoseiidae) on almond pollen for up to 20 generations did not negatively impact its life table parameters [61]. Similarly, *N. cucumeris* reared on almond pollen for over 50 generations did not exhibit any decline in fitness [54,65]. Furthermore, in *Amblyseius orientalis*, where mass rearing on factitious food for five generations resulted in a significant decline in predator performance, switching from the factitious food to natural prey for only two generations was sufficient to reverse the effects [51,52]. Therefore, future studies should extend to at least 10–20 generations.

While natural prey such as *T. urticae* may offer a more complete nutrient profile or induce prey-handling behavior, their use in mass rearing is constrained by logistics and economic challenges. In contrast, frozen *C. lactis* and pollen-based diets offer consistent quality, lower pathogen risk, and easier scaling for commercial production [39,46,50]. The observed high reproductive performance on these foods, coupled with no apparent trade-offs in prey-handling ability or fecundity, makes them promising candidates for either small scale laboratory or commercial rearing of *A. andersoni*.

The ability to sustain populations on factitious foods like *C. lactis* and cattail pollen can be exploited to maintain predator populations during prey scarcity, thus enhancing persistence and reliability of biocontrol [27,53]. Furthermore, it could result in fewer release events, reduced labor costs, and more stable predator population dynamics. Additionally, the lack of differences in performance between individuals reared on natural vs. factitious diets supports the feasibility of producing robust predators without compromising field effectiveness.

A broader ecological implication of our findings relates to the generalist feeding behavior of *A. andersoni*, which appears to confer high adaptability to diverse food resources. This trait is advantageous for biological control, as it supports resilience to environmental variability and prey scarcity. Compared to specialized phytoseiids like *Phytoseiulus persimilis* Athias-Henriot (Acari: Phytoseiidae), which rely almost exclusively on *T. urticae* for survival and reproduction, generalist predators such as *A. andersoni* are better suited for preventive control strategies, as their ability to utilize a variety of alternative food sources—such as pollen or factitious prey facilitates earlier establishment and persistence in the crop, even in the absence of target pests [17].

## 4. Materials and Methods

### 4.1. Predator Rearing

A laboratory colony of *A. andersoni* was established using approximately 50 individuals collected by leaf sampling in July 2023 from tomato fields in the area of Messenia (37°00′26.8” N 21°40′17.2” E). The mites were collected as part of an extensive field sampling effort conducted across continental Greece, aimed at identifying phytoseiid mites associated with cultivated tomato fields and wild plants bearing glandular trichomes. The colony was maintained on Plexiglas placed on wet cotton in plastic cups, as described by Koveos and Broufas [28]. Along with cattail pollen (Nutrimite™), twice per week, spider mite-infested tomato leaves were provided as prey for the mites.

### 4.2. Plants

Tomato (*Solanum lycopersicum* L., cv. Moneymaker) plants were grown from seeds in peat in plastic pots (350 mL). The plants were grown in a climate room at 25 ± 1 °C, 60–80%RH and 16:8 LD. Water was supplied every other day. The light source consisted of LED tubes (3 and 1 tube of 5000 and 3000 K, respectively), providing a light intensity of approx. 27,000 lux at the plant canopy level. As a growth substrate, fertilized peat (TS2, Klasmann) was used. After the second week, following transplanting, tomato plants were irrigated weekly till runoff with a water-soluble fertilizer solution (NPK 10-10-10) at a rate of 1 g L^−1^.

In the bioassays, leaflets from plants with three fully expanded leaves (21 days after transplanting) were used. Water was provided every other day.

### 4.3. Experimental Arenas

Tomato leaf discs (ø 4 cm) cut from fully grown leaves of 4–5 weeks old plants, or plastic Plexiglas plates (approx. 3.5 × 4 cm) were used as experimental arenas. These were placed with their adaxial side in contact with a water-soaked cotton layer in plastic Petri dishes (ø 5 cm). On each rearing unit, a plastic sheet (5 × 5 mm, 1 mm thick) was placed as shelter for the mites.

### 4.4. Effects of Different Substrates and Food Sources on Predator Performance

#### 4.4.1. Food Sources

Pollen grains of maize (*Zea mays* L. (Gramineae)), pine (*Pinus nigra* J.F. Arnold (Pinaceae)), olive (*Olea europea* L. (Oleaceae)), cattail (*T. angustifolia* L.), or frozen astigmatic *C. lactis* mites were used as food sources, after mite larvae hatching. All pollens, besides the commercial one (Nutrimite™), were collected from the field following the procedures described by Koveos and Broufas [28] and maintained at −20 °C. Briefly, for pollen collection, freshly collected flowers or male structures were bagged and shaken to release pollen grains. The collected pollen was then air-dried for 12 h indoors at room temperature, 25 ± 1 °C and low humidity ~40% and subsequently sieved (100 mesh) to remove debris, and stored in glass vials at −20 °C until use (approx. 6–8 months). The colony of *C. lactis* was maintained on a black Plexiglas plate positioned at the top of a sponge within a water-filled plastic container. The container lid was perforated with small openings covered with a 150-mesh fabric to facilitate adequate ventilation and air exchange. The colony was inspected every other day, and fresh baker’s yeast and water were supplied as necessary. The colony was maintained at a temperature of 25 ± 2 °C. For the experiments, mixed developmental stages of *C. lactis* were transferred into 0.5 mL Eppendorf tubes using a fine hairbrush (No. 000) and then frozen at −20 °C. The frozen mites were stored at this temperature for up to one week before use in the experiments. Before introduced to the rearing units of the predatory mite, the frozen mites were thawed at room temperature for approx. fifteen minutes, allowing them to reach ambient temperature.

#### 4.4.2. Juvenile Development, Survival, and Oviposition of *A. andersoni* on Different Diets

Cohorts of predatory mite eggs were obtained by allowing young (6–8 days old) *A. andersoni* females from the stock colony to lay eggs for 12 h. The eggs were subsequently placed individually on the experimental arenas and inspected twice daily. The survival and developmental stage of each predator was recorded until adulthood. For each treatment, 13–17 replicates were used. Upon adult emergence, the sex of the individuals was recorded, and the mites were transferred in pairs to new arenas with the same diet (food source) on which they were reared during juvenile development. Survival and oviposition were recorded daily for one week starting from the onset of oviposition. For each treatment, 13–17 replicates (individuals on the leaf substrate: *Z. mays*: 15; *O. europea*: 14; *P. nigra*: 14; *T. angustifolia*: 15; *C. lactis*: 16, individuals on the plexiglas substrate: *Z. mays*: 16, *O. europea*: 16; *P. nigra*: 13; *T. angustifolia*: 17; *C. lactis*: 17—the number of replicates per treatment were comparable with reported values in similar studies [66,67] and are considered sufficient given the high number of treatments conducted simultaneously and the low within-treatment variability observed) were used.

Throughout the experiments, the food offered to the predators was replaced daily with pollen grains provided at approx 0.3 mg/cm^2^, whereas *C. lactis* was offered ad libitum. To maintain a constant and standardized food supply daily, the unconsumed pollen and prey individuals were carefully removed and replaced. For nine consecutive days, the arenas were inspected, and eggs laid by each female were recorded, collected, and transferred to new rearing units. The resulting larvae were reared to adulthood on the same food source as their parents, and the emerged adults were sexed to determine the sex ratio of the offspring. Experiments were conducted at 25 ± 1 °C, 16:8 LD and 60–70% RH. Arenas were randomized across treatments to minimize bias. Treatments included two substrates (leaf disc vs. Plexiglas plate) and five food sources (pollen and frozen *C. lactis*).

### 4.5. Mass-Rearing Effects on Predator Performance

#### Predator Fitness

Fitness traits of *A. andersoni* were evaluated for two populations that were continuously reared for 5 consecutive generations on Plexiglas (no effect of the rearing substrate on the performance of *A. andersoni* was recorded in the first group of experiments): one, fed with a mixture of the best performing food sources (i.e., cattail pollen plus frozen *C. lactis*), and one fed with spider mites plus cattail pollen. Experiments were conducted as above, and fitness components evaluated included for both populations demographic parameters (developmental time, juvenile survival, oviposition, progeny sex ratio, as well as the intrinsic rates of increase) and predation efficiency (daily prey consumption and respective oviposition).

Experimental arenas consisted of tomato leaf discs (Ø 4 cm) placed on a moist cotton layer in Petri dishes to maintain humidity and prevent mite escape. For *T. urticae*, ten adult females were introduced onto each leaf disc and allowed to oviposit for 24 h. After this period, the adults were carefully removed with the help of a fine entomological needle to minimize disturbance to the webbing. Once the spider mite eggs hatched, larval density was standardized to 40 individuals per arena by removing excess larvae using the same method.

For *Aculops lycopersici* (Tryon) (Acari: Eriophyidae), 90 individuals were transferred to each leaf disc using a fine camel-hair brush. Eggs of the predatory mite laid within 12 h were individually transferred on the experimental arenas. Upon hatching of the young larvae, experimental arenas were inspected daily until the emergence of adults. During this period, the survival of the predators and their developmental stage were recorded. The prey individuals consumed each day were replaced to ensure a continuous food supply for the predator. In the pollen treatment, old pollen grains were removed daily and replaced with fresh.

Following adult emergence, the same procedure was continued to assess adult survival, daily oviposition, and offspring sex ratio. Observations were conducted daily for up to seven days after the preoviposition period.

In the predation experiments, a young adult female predator (4–6 days old) from each of the two rearing systems was individually introduced into an experimental arena containing prey (*T. urticae* or *A. lycopersici*) as described above. Prior to the experiment, all predator females were starved for 24 h by placing them on water-soaked Plexiglas plates, which provided access to water while preventing escape. Following the introduction of the predators into the experimental units, prey consumption and oviposition were recorded 24 h later. All assays were conducted under controlled conditions at 25 ± 2 °C, 16:8 LD, and 60–70% RH. Arenas were randomized across treatments to minimize bias, and 10 replicates were used per treatment.

### 4.6. Intrinsic Rates of Population Increase (r_m_)

Calculations of the intrinsic rates of population increase (*r_m_*) of *A. andersoni* in the different treatments were performed by solving the equation [68]:

*r_m_* = (net reproductive rate) × exp((-*r_m_*) × (egg-to-egg period))

where the net reproductive rate equals (peak oviposition rate) × (survival in egg-to-egg period) × (offspring’s sex ratio), as described in Nomikou et al. [69].

### 4.7. Statistical Analysis

To evaluate the effects of key variables, a Generalized Linear Model (GLM) was applied. The model assessed the influence of three fixed factors: food source (pollens and frozen *C. lactis*), sex (male vs. female), and rearing substrate (leaf vs. Plexiglas), and their interactions on two response variables: juvenile developmental time and cumulative female oviposition over 7 days (in the latter case only the food source and the type of the rearing substrate were evaluated).

A full factorial model was used with the GLM fitted using a normal distribution with an identity link function, or Poisson distribution with a log link function for the analyses of developmental time and cumulative oviposition, respectively. The same approach was used in the second group of experiments, in which we evaluated the effect of two mass rearing systems based on the use of cattail pollen as a food source, supplemented by prey individuals of either the two-spotted spider mite or frozen *C. lactis*. A GLM with a normal distribution and identity link function was used to assess the effects on juvenile developmental time.

The Wald chi-square test was used to assess the statistical significance of main effects and interaction terms. Where significant main effects were identified, pairwise comparisons of estimated marginal means were conducted using the Least Significant Difference (LSD) method to determine specific group-level differences. A Student *t*-test was used to compare the mean cumulative oviposition within each tested food source. To evaluate differences in juvenile survival rates among treatments, a Chi-square (χ^2^) test was used to compare the proportions of surviving versus non-surviving individuals across treatment groups, based on categorical survival data. The Student *t*-test was used to compare the mean daily prey consumption and oviposition of females reared under the two mass rearing protocols. All statistics were performed using IBM SPSS Statistics 30.

## 5. Conclusions

In conclusion, this study confirms the high suitability of cattail pollen and *C. lactis* as food sources for *A. andersoni*, with no short-term negative effects from factitious diet rearing. These findings have important implications for cost-effective, scalable mass production and for improving the sustainability of augmentative biological control strategies. Future work should extend rearing duration, test predator performance under semi-field conditions, and investigate physiological and behavioral traits over longer generational spans.

## Data Availability

Data are contained within the article.
